# Supplementary material for: The interactome of intact mitochondria by cross-linking mass spectrometry provides evidence for coexisting respiratory supercomplexes
Source: Mol Cell Proteomics. 2017 Dec 8;17(2):216–32. doi: 10.1074/mcp.RA117.000470 (PMC5795388; doi:10.1074/mcp.RA117.000470)
Supplement: Supplemental Data [file supp_RA117.000470_133922_0_supp_23978_fzffwf.zip › spectra_annotation/mito_DR_spectra_annotation/85-1-14-1-5-1.pdf]

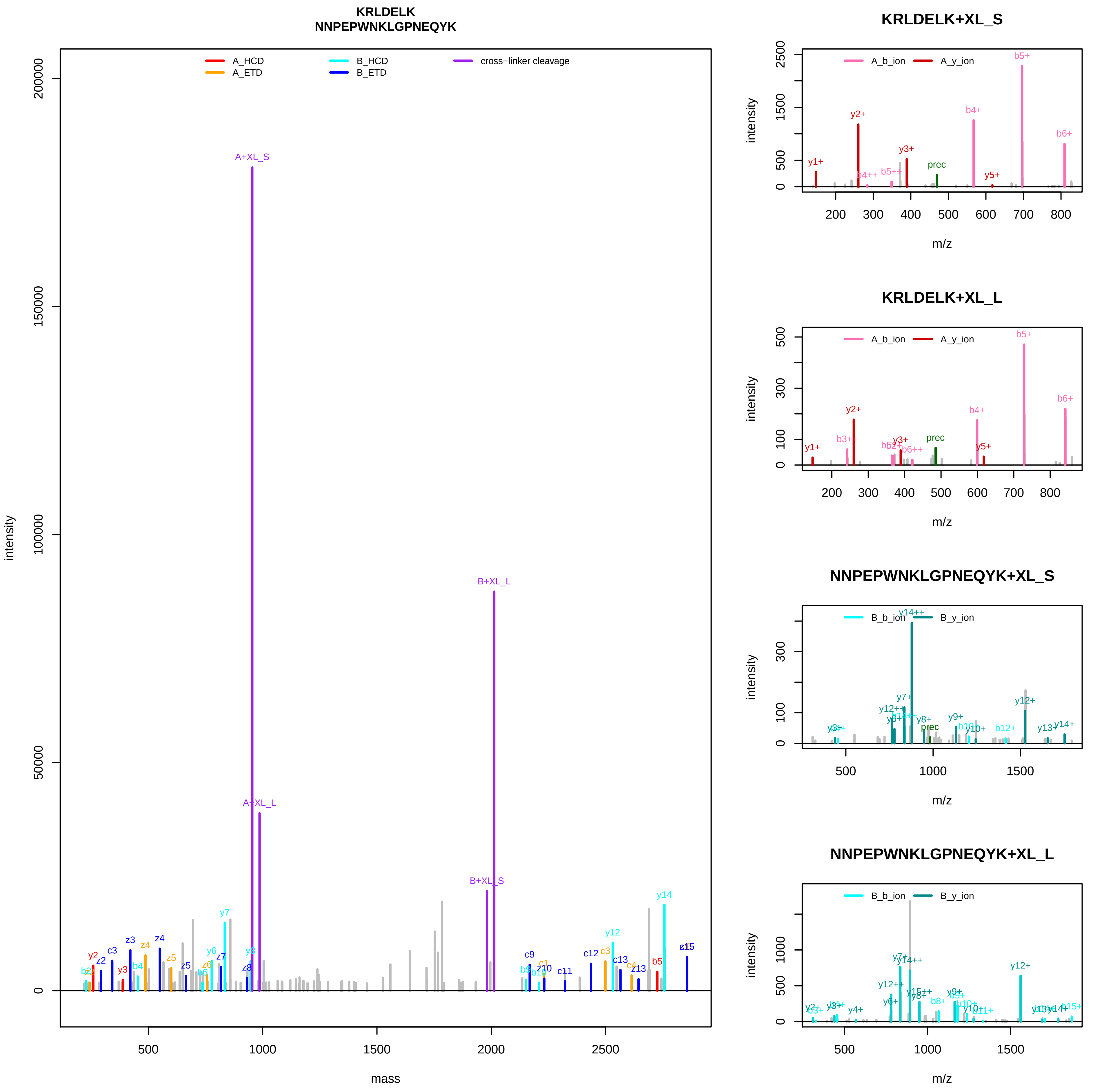

KRLDELK
NNPEPWNKLGPNEQYK
A_HCD
A_ETD
B_HCD
B_ETD
cross−linker cleavage
A+XL_S
A+XL_L
B+XL_L
B+XL_S
y2
y3
c3
z2
z3
z4
b4
z5
z6
y6
y7
z7
z8
y8
c9
c10
z10
c11
c12
c3
y12
c13
c4
z13
b5
y14
z15
c15
intensity
mass
0
50000
100000
150000
200000
500
1000
1500
2000
2500
KRLDELK+XL_S
A_b_ion
A_y_ion
y1+
y2+
b4++
b5++
y3+
prec
b4+
y5+
b5+
b6+
intensity
m/z
0
500
1500
2500
200
300
400
500
600
700
800
KRLDELK+XL_L
y1+
y2+
b3++
y3+
b6++
prec
b4+
y5+
b5+
b6+
0
100
300
500
NNPEPWNKLGPNEQYK+XL_S
B_b_ion
B_y_ion
y14++
y7+
y12++
y8+
prec
y9+
y10+
b12+
y12+
y13+
y14+
0
100
300
1000
1500
NNPEPWNKLGPNEQYK+XL_L
y7+
y14++
y12++
y6+
y9+
y10+
b8+
y12+
y4+
y3+
y2+
y13+
y14+
b15+
0
500
1000
